# The Usage of Human IGHJ Genes Follows a Particular Non-random Selection: The Recombination Signal Sequence May Affect the Usage of Human IGHJ Genes

**DOI:** 10.3389/fgene.2020.524413

**Published:** 2020-12-08

**Authors:** Bin Shi, Xiaoheng Dong, Qingqing Ma, Suhong Sun, Long Ma, Jiang Yu, Xiaomei Wang, Juan Pan, Xiaoyan He, Danhua Su, Xinsheng Yao

**Affiliations:** ^1^Department of Immunology, Center of Immunomolecular Engineering, Innovation & Practice Base for Graduate Students Education, Zunyi Medical University, Zunyi, China; ^2^Department of Breast Surgery, Affiliated Hospital of Zunyi Medical University, Zunyi, China; ^3^Department of Laboratory Medicine, Affiliated Hospital of Zunyi Medical University, Zunyi, China; ^4^School of Laboratory Medicine, Zunyi Medical University, Zunyi, China

**Keywords:** High-throughput sequencing, V(D)J, IGHJ, “12/23” rule, BCR

## Abstract

The formation of the B cell receptor (BCR) heavy chain variable region is derived from the germline V(D)J gene rearrangement according to the “12/23” rule and the “beyond 12/23” rule. The usage frequency of each V(D)J gene in the peripheral BCR repertoires is related to the initial recombination, self-tolerance selection, and the clonal proliferative response. However, their specific differences and possible mechanisms are still unknown. We analyzed in-frame and out-of-frame BCR-H repertoires from human samples with normal physiological and various pathological conditions by high-throughput sequencing. Our results showed that IGHJ gene frequency follows a similar pattern which is previously known, where IGHJ4 is used at high frequency (>40%), IGHJ6/IGHJ3/IGHJ5 is used at medium frequencies (10∼20%), and IGH2/IGHJ1 is used at low frequency (<4%) under whether normal physiological or various pathological conditions. However, our analysis of the recombination signal sequences suggested that the conserved non-amer and heptamer and certain 23 bp spacer length may affect the initial IGHD-IGHJ recombination, which results in different frequencies of IGHJ genes among the initial BCR-H repertoire. Based on this “initial repertoire,” we recommend that re-evaluation and further investigation are needed when analyzing the significance and mechanism of IGHJ gene frequency in self-tolerance selection and the clonal proliferative response.

## Introduction

The diversity of the initial vertebrate B cell receptor (BCR) originates from the recombination of multiple germline genes (V(D)J) and insertion and deletion during the recombination process. There is a consensus recombination signal sequence (RSS; Sakano et al., 1979) at the 5′ or 3′ end of each V(D)J gene segment that participates in recombination according to the “12/23” rule (Tonegawa, 1983; Hesse et al., 1987; Lewis, 1994) and the “beyond 12/23” rule (Bassing et al., 2000). In addition, recombination-activating gene (RAG) enzymes, terminal deoxynucleotidyl transferase (TDT), heterodimer-KU70/KU80, DNA-dependent protein kinase (DNA-PK/Artemis), DNA ligase IV (XRCC4), and other proteins are involved in the complex V(D)J recombination process (Lewis, 1994; Bogue and Roth, 1996). Recently, a research reported cryoelectron microscopy structures of synaptic RAG complexes at up to 3.4 Å resolution, which reveal a closed conformation with base flipping and base-specific recognition of RSSs (Ru et al., 2015). Another study employed a single-molecule method to track the RAG–RSS interaction, which can provide a relatived complete kinetic description of the initial phases of V(D)J recombination (Hirokawa et al., 2020).

Theoretically, the usage frequency of V(D)J gene segments is random in the pro-B cell or pre-B cell recombination process (before autoantigen selection). However, *in vitro* experiments in B cell lines confirmed that V(D)J gene segments contribute unequally to the primary repertoire, and the consensus heptamer and non-amer sequences of the RSSs are considered a major factor (Feeney et al., 2000). The contributing factors may also relate to the usage frequency of V(D)J gene segments. The usage of proximal and distal gene segments in recombination is not random; for example, the JH-proximal VH gene of pre-B cell lines has a preferential usage (Yancopoulos et al., 1984), and VH near Cu may be preferred during early rearrangement (Perlmutter et al., 1985). During pre-B cell differentiation and development, the initial DH-JH rearrangements employ more 3′ (JH-proximal) DH segments (Reth et al., 1986); however, Feeney et al. (1997) found that there is no apparent preference for the more JK-proximal over the more JK-distal genes in the proximal region. In addition, compared with RSSs with one or more base mutations, the corresponding gene subfamily of RSSs with a consensus heptamer/non-amer (conserved) has preferred usage (Hesse et al., 1987; Akamatsu et al., 1994; Ramsden et al., 1996; Steen et al., 1997; Larijani et al., 1999). Moreover, the usage frequency of the corresponding gene segment will be affected when the lengths of the 12 bp spacer/23 bp spacer in RSSs increase or decrease (Akamatsu et al., 1994; Ramsden et al., 1996; Steen et al., 1997) and when the base sequences of the 12 bp spacer/23 bp spacer in RSS change (Fanning et al., 1996; Nadel et al., 1998; Montalbano et al., 2003). In addition, expression level of transcription factors and changes in chromatin structure may also influence individual V gene rearrangement frequency (Feeney et al., 2004).

However, these results are derived from experiments based on B cell lines *in vitro*, and whether RSSs influence the V(D)J usage frequency of initial repertoires *in vivo* is unclear. The difference in each V(D)J usage frequency in the peripheral B cell repertoires is mainly derived from the selection of self-tolerance and the response of clonal proliferation (Yancopoulos et al., 1984; Gu et al., 1991; Groettrup and von Boehmer, 1993; Ten Boekel et al., 1997). How the difference in usage frequency of each V(D)J gene segment in initial repertoires influences the peripheral repertoire has not been clarified and has received little attention.

With the development of next-generation sequencing (NGS) analysis for V(D)J tracking, analyzing each V(D)J usage frequency in individual BCR-H repertoires is now possible. Since 2013, we have broadly analyzed the composition characteristics of the BCR-H repertoires through high-throughput sequencing (HTS) and found that the human IGHJ4 gene has the highest usage frequency whether under physiological or various pathological conditions, followed by IGHJ6, IGHJ3, and IGHJ5 with medium usage frequency and by IGHJ1 and IGHJ2 with significantly low usage frequency (Supplementary Table 1). Additionally, the usage frequency of 6 IGHJ gene families shows amazing consistency by analyzing the BCR-H sequences of public databases (IMGT, etc.) and published articles (HTS data) from subjects with physiological or various pathological conditions. Specifically, we analyzed the composition characteristics of the RSSs in human IGHJ genes. Our results suggest that the consensus non-amer and heptamer, the standard spacer length (23 bp), and the mutation site of RSSs may affect the usage frequency of 6 IGHJ gene segments (non-random selection), and this specific primary repertoire may result in the lack of significant changes in the usage frequency of 6 IGHJ genes in the peripheral repertoire under normal physiological and various pathological conditions.

## Results

### The IGHJ Gene Frequency Follows a Particular Non-random Selection

**FIGURE 1:**
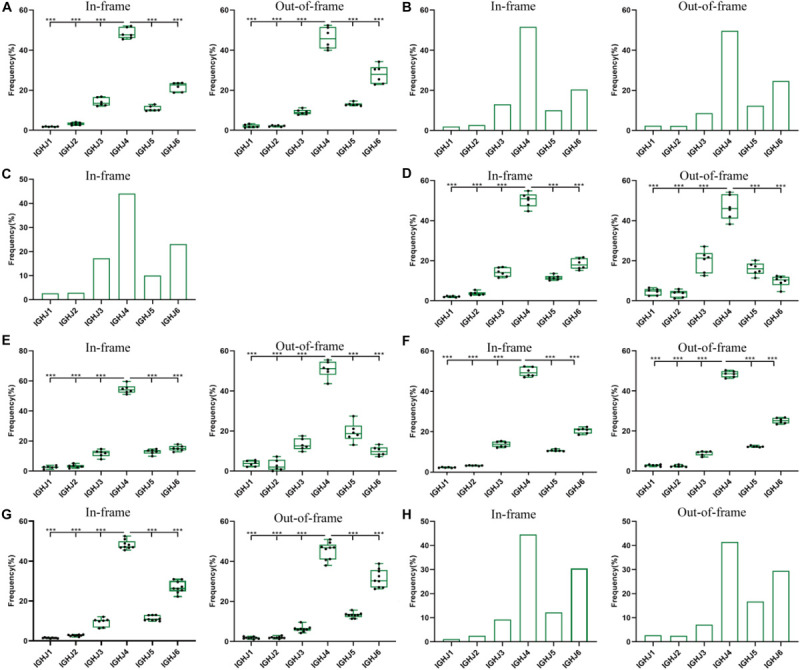
The usage frequencies of 6 IGHJ genes in the in-frame and out-of-frame BCR-H repertoire from different subjects. **(A)** The IGHJ usages of BCR-H repertoire from 6 Healthy volunteers. **(B)** The IGHJ usages of BCR-H repertoire from public data. **(C)** The IGHJ usages of BCR-H sequences (*n* = 9430) from IMGT data. **(D)** The IGHJ usages of IgM-H repertoire from volunteers before and after immunization with the HBV vaccine. **(E)** The IGHJ usages of IgG-H repertoire from volunteers before and after immunization with the HBV vaccine. **(F)** The IGHJ usages of BCR-H repertoire from SLE volunteers. **(G)** The IGHJ usages of BCR-H repertoire from breast cancer volunteers. **(H)** The IGHJ usages of BCR-H repertoire from volunteers with a high titer of HbsAb. We used bar chart for data of sample groups with *n* = 2 **(B,H)** and IMGT **(C)**, while used box plot for data of sample groups with *n* ≥ 3 by a one-way ANOVA with Bonferroni correction **(A,D–G)**. All statistically significant differences are indicated as *p < 0.05; **p < 0.01, and ***p < 0.001.

**FIGURE 2:**
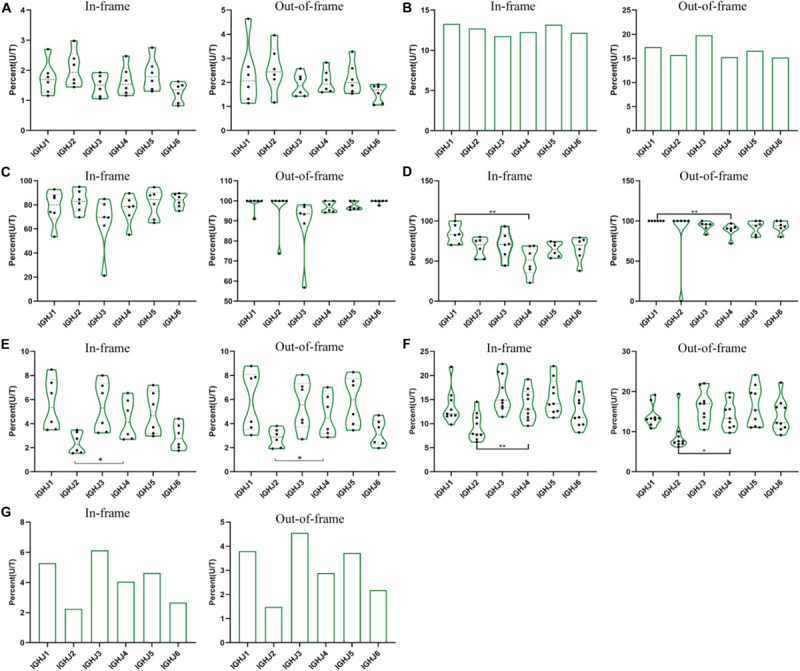
The ratio of unique to total sequences (U/T) of 6 IGHJ genes in the in-frame and out-of-frame BCR-H repertoires from different subjects. **(A)** The IGHJ U/T ratio of BCR-H repertoires from 6 Healthy volunteers. **(B)** The IGHJ U/T ratio of BCR-H repertoires from public data. **(C)** The IGHJ U/T ratio of IgM-H repertoires from volunteers before and after immunization with the HBV vaccine. **(D)** The IGHJ U/T ratio of IgG-H repertoires from volunteers before and after immunization with the HBV vaccine. **(E)** The IGHJ U/T ratio of BCR-H repertoires from SLE volunteers. **(F)** The IGHJ U/T ratio of BCR-H repertoires from breast cancer volunteers. **(G)** The IGHJ U/T ratio of BCR-H repertoires from volunteers with a high titer of HbsAb. We used bar chart for sample groups with *n* = 2 **(B)** and IMGT **(C)**, while used box plot for sample groups with *n* ≥ 3 using a one-way ANOVA with Bonferroni correction **(A,D–G)**. All statistically significant differences are indicated as *p < 0.05; **p < 0.01, and ***p < 0.001.

**FIGURE 3:**
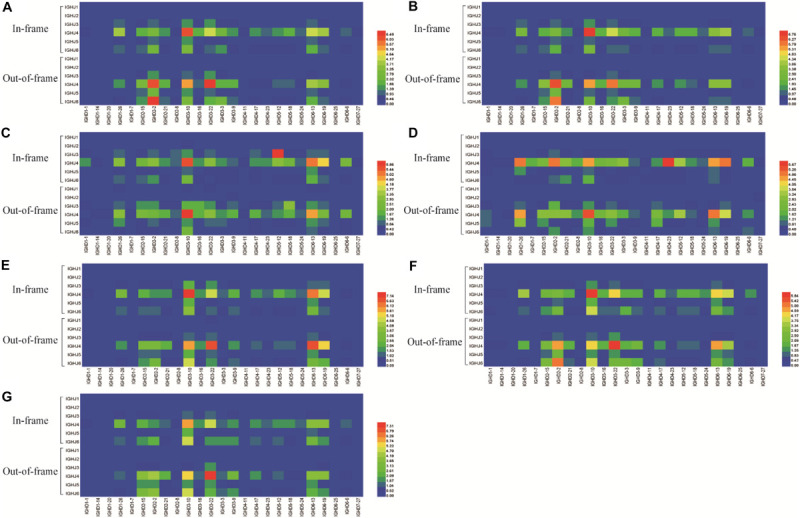
IGHJ-IGHD pairing in the in-frame and out-of-frame BCR-H repertoires from different subjects. **(A)** IGHJ-IGHD pairing of BCR-H repertoires from 6 Healthy volunteers. **(B)** IGHJ-IGHD pairing of BCR-H repertoires from public data. **(C)** IGHJ-IGHD pairing of IgM-H repertoires from volunteers before and after immunization with the HBV vaccine. **(D)** IGHJ-IGHD pairing of IgG-H repertoires from volunteers before and after immunization with the HBV vaccine. **(E)** IGHJ-IGHD pairing of BCR-H repertoires from SLE volunteers. **(F)** IGHJ-IGHD pairing of BCR-H repertoires from breast cancer volunteers. **(G)** IGHJ-IGHD pairing of BCR-H repertoires from volunteers with a high titer of HbsAb.

We analyzed the usage frequencies of IGHJ genes from 8 groups of data (Figure 1 and Supplementary Tables 1–7). Among them, except for data 2 and 3 (Figures 2, 3 and Supplementary Tables 2, 3) from public data, the other groups of data are from our laboratory. The number of BCR-H sequences from 6 healthy volunteer samples ranged from 250,000 to 1,250,000 (Supplementary Table 1). The order of frequency of IGHJ genes (in-frame) was IGHJ4 > IGHJ6 > IGHJ3 > IGHJ5 > IGHJ2 > IGHJ1, while out-of-frame sequences followed an order of IGHJ4 > IGHJ6 > IGHJ5 > IGHJ3 > IGHJ1 > IGHJ2 (Figure 1A and Supplementary Table 1). For these two groups, the frequency of IGHJ4 was significantly higher than that of each IGHJ gene, while IGHJ1 and IGHJ2 were significantly less frequently used (Figure 1A and Supplementary Table 1). Supplementary Table 2 shows the data of the naive B cell repertoire (primary repertoire, *n* = 48,167) and the memory B cell repertoire (*n* = 50,290). The order of IGHJ gene usage (out-of-frame) was IGHJ4 > IGHJ6 > IGHJ5 > IGHJ3 > IGHJ1 > IGHJ2 (Figure 1B and Supplementary Table 2), while the usage of IGHJ genes (in-frame) followed IGHJ4 > IGHJ6 > IGHJ3 > IGHJ5 > IGHJ2 > IGHJ1. Sequences (*n* = 9,340) from the IMGT/LIGM-DB also followed this pattern (Figure 1C and Supplementary Table 3). Similarly, IGHJ4 was significantly used, while the IGHJ1 or IGHJ2 frequency was significantly lower than those of other IGHJ genes.

A similar pattern of IGHJ gene frequency was found not only under normal physiological conditions but also under pathological conditions. IgM and IgG sequences from three volunteers before and after HBV vaccine are shown in Supplementary Table 4. IgM in-frame sequences presented as IGHJ4 > IGHJ6 > IGHJ3 > IGHJ5 > IGHJ2 > IGHJ1, while IgM out-of-frame sequences showed IGHJ4 > IGHJ3 > IGHJ5 > IGHJ6 > IGHJ1 > IGHJ2 (Figure 1D and Supplementary Table 4). For IgG sequences, IGHJ4 > IGHJ6 > IGHJ5 > IGHJ3 > IGHJ2 > IGHJ1 was found in the in-frame sequences, while out-of-frame sequences showed IGHJ4 > IGHJ5 > IGHJ3 > IGHJ6 > IGHJ1 > IGHJ2 (Figure 1E and Supplementary Table 5). The BCR-H sequences from 6 SLE samples ranged from 170,000 to 610,000 sequences (Supplementary Table 5). The usage frequency of 6 IGHJ genes (in-frame) followed IGHJ4 > IGHJ6 > IGHJ3 > IGHJ5 > IGHJ2 > IGHJ1, while the order of usage frequency of 6 IGHJ genes (out-of-frame) was IGHJ4 > IGHJ6 > IGHJ5 > IGHJ3 > IGHJ1 > IGHJ2 (Figure 1F and Supplementary Table 6). The BCR-H sequence number from breast cancer samples was approximately 70,000∼160,000 for each sample (Supplementary Table 6), and the sequence number from two volunteers with a high titer of HBsAb was 760,000 and 880,000 (Supplementary Table 7). Interestingly, in-frame and out-of-frame sequences from these two groups consistently presented as IGHJ4 > IGHJ6 > IGHJ5 > IGHJ3 > IGHJ2 > IGHJ1 (Figures 1G,H and Supplementary Table 8).

In addition, we analyzed the ratio of unique to total sequences of each IGHJ gene (in-frame and out-of-frame) and found no differences in 6 IGHJ gene families (Supplementary Tables 1, 2, and Figure 2), which suggests that the multiplex PCR library and the experimental system of HTS did not show obvious bias. Taken together, these results indicate that IGHJ gene frequency follows a similar pattern where IGHJ4 is used at high frequency (>40%), IGHJ6/IGHJ3/IGHJ5 is used at medium frequencies (10∼20%), and IGH2/IGHJ1 is used at low frequencies (<4%). Therefore, the pattern shows high consistency in normal physiological and various pathological conditions, which suggests that the recombination selection of each IGHJ gene is a particular non-random pattern.

### IGHJ-IGHD Pairing and Trimming and Insertion Between IGHD and IGHJ

**FIGURE 4:**
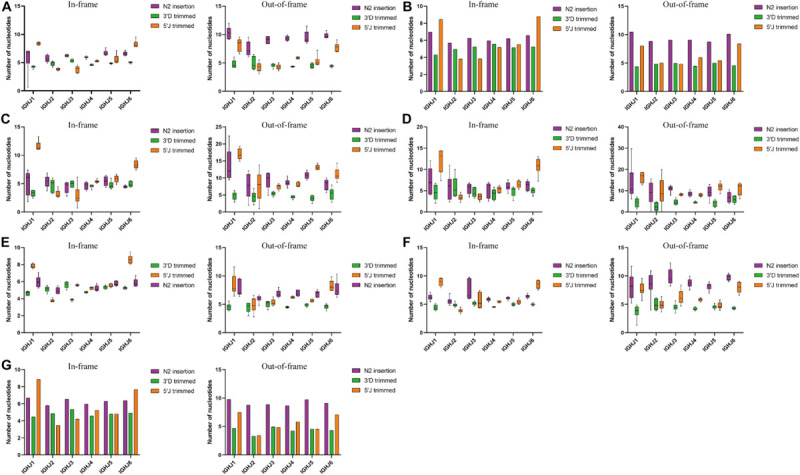
3′D trimmed, 5′J trimmed and N2 insertion at IGHD-IGHJ junction in the in frame and out of frame BCR-H repertoires from different subjects. **(A)** BCR-H repertoires from 6 Healthy volunteers. **(B)** BCR-H repertoires from public data. **(C)** IgM-H repertoires from volunteers before and after immunization with the HBV vaccine. **(D)** IgG-H repertoires from volunteers before and after immunization with the HBV vaccine. **(E)** BCR-H repertoires from SLE volunteers. **(F)** BCR-H repertoires from breast cancer volunteers. **(G)** BCR-H repertoires from volunteers with a high titer of HbsAb. We used bar chart for data of sample groups with *n* = 2 **(B)** and IMGT **(C)**, while used box plot for data of sample groups with *n* ≥ 3 by a one-way ANOVA with Bonferroni correction **(A,D–G)**.

Six IGHJ gene families have different initial BCR-H repertoires, which may be related to non-random selection of D-J recombination, thus prompting us to investigate IGHJ-IGHD pairing (Figure 3) and trimming and insertion between IGHD and IGHJ (Figure 4). Most of the 27 IGHD gene subfamilies showed a higher proportion of IGHJ4 pairing (Figure 3). However, whether they were in frame or out of frame, the paired IGHD genes of different IGHJ genes at high or low frequencies were similar. For 6 IGHJ gene families, the IGHD genes paired at high frequency included IGHD6-13, IGHD6-19, IGHD3-22, IGHD3-10, and IGHD2-15, while the low frequency parings included IGHD1-20, IGHD1-7, IGHD4-11, IGHD6-25, and IGHD7-27 (Figure 3).

Trimming and insertion between IGHD and IGHJ mainly presented as 3′D trimmed, 5′J trimmed, and N2 insertion (Figure 4). We found that the mean length of 5′J trimmed showed significant differences among different IGHJ genes under some conditions, while 3′D trimmed and N2 insertion did not show significant differences (data not shown). For IGHJ1 and IGHJ2, the 5′J trimmed length of IGHJ1 (in-frame sequences) showed significant differences compared with the other IGHJ subfamilies in the SLE and IgM with HBV vaccine groups (one-way ANOVA with Bonferroni correction, *p* < 0.001). A similar situation occurred on 5′J trimmed of IGHJ2 in the breast cancer group. The mean length of 5′J trimmed of the IGHJ4 (in-frame or out-of-frame sequences) showed significant differences compared with the other IGHJ genes in the SLE group (one-way ANOVA with Bonferroni correction, each *p* < 0.001). In all groups, IGHJ4 (high usage) showed significant differences compared with IGH1 and IGHJ2 (low usage; one-way ANOVA with Bonferroni correction, each *p* < 0.001). The mean length of 5′J trimmed from IGHJ6/IGHJ5/IGHJ3 (in-frame sequences) showed significant differences compared with that of the other 5 IGHJ subfamilies in different groups (one-way ANOVA with Bonferroni correction, each *p* < 0.001). These results suggest that the composition of the IGHJ front end (5′J trimmed) may have an impact on the usage and efficiency of the D-J recombination, especially for the IGHJ genes with high or low usage.

### The Usage Frequency of 6 IGHJ Families in the BCR-H Repertoires From Public Data

We analyzed the usage frequency of the 6 IGHJ gene families in BCR-H repertoires from 19 published articles (Forconi et al., 2010; Racanelli et al., 2011; Ippolito et al., 2012; Prabakaran et al., 2012; Yin et al., 2013; Briney et al., 2014; Mroczek et al., 2014; Lecerf et al., 2015; Martin et al., 2015, 2016; Zhang et al., 2015, 2017; Guo et al., 2016; Kerzel et al., 2016; Rother et al., 2016; Tan et al., 2016; Liu et al., 2017; Roy et al., 2017; Hirokawa et al., 2019; Supplementary Table 8). Overall, subjects included healthy volunteers of different ages (2 months to 87 years) and patients with different pathological conditions, including SLE, primary biliary cholangitis (PBC), colorectal adenoma and carcinoma (CRC), celiac disease (CD), congenital heart disease, atopic dermatitis, hepatitis C virus infection, rheumatoid arthritis, and primary immune thrombocytopenia, as well as in humanized NOD-scid-IL2R gamma (null) mice. The sample sources included peripheral blood, PBMC (DNA), PBMC (RNA), cord blood, biopsies (RNA), humanized mouse spleen, bone marrow, mucosal tissues, small intestine, lung, stomach, lymph node, tonsil, and thymus. The B cell subsets included B cells, pre-B cells, immature B cells, transitional B cells, naive B cells, normal B cells with IGHV1-69-DJ-C rearrangements, memory B cells, and plasmacytes, etc.

The usage frequency of the IGHJ4 gene subfamily was higher than that of other IGHJ genes, suggesting that IGHJ4 had the highest frequency in the initial rearrangement and showed high consistency in peripheral repertoires (after self-tolerance selection or the clonal proliferation response). The usage frequencies of IGHJ1 and IGHJ2 were significantly lower than those of the other IGHJ genes, suggesting that IGHJ1 and IGHJ2 may be partially restricted in the initial rearrangement and that they showed consistency in the peripheral repertoires. IGHJ6, IGHJ3, and IGHJ5 have a medium usage frequency, and the usage frequency of IGHJ6 was higher than that of IGH3 and IGHJ5, except for articles (Akamatsu and Oettinger, 1998; Briney et al., 2014; Mroczek et al., 2014; Murphy et al., 2014; Zhang et al., 2015). Additional results showed that IGHJ3 usage was higher than IGHJ5. Regardless of the physiological or pathological conditions, the usage frequencies of the 6 IGHJ gene families in our results are almost identical to those in the 19 published articles. The overall results indicate the non-randomness of the 6 human IGHJ gene usages during the initial rearrangement process.

### IGHD-IGHJ Recombination May Affect IGHJ Gene Usage Through the RSS Composition

**FIGURE 5:**
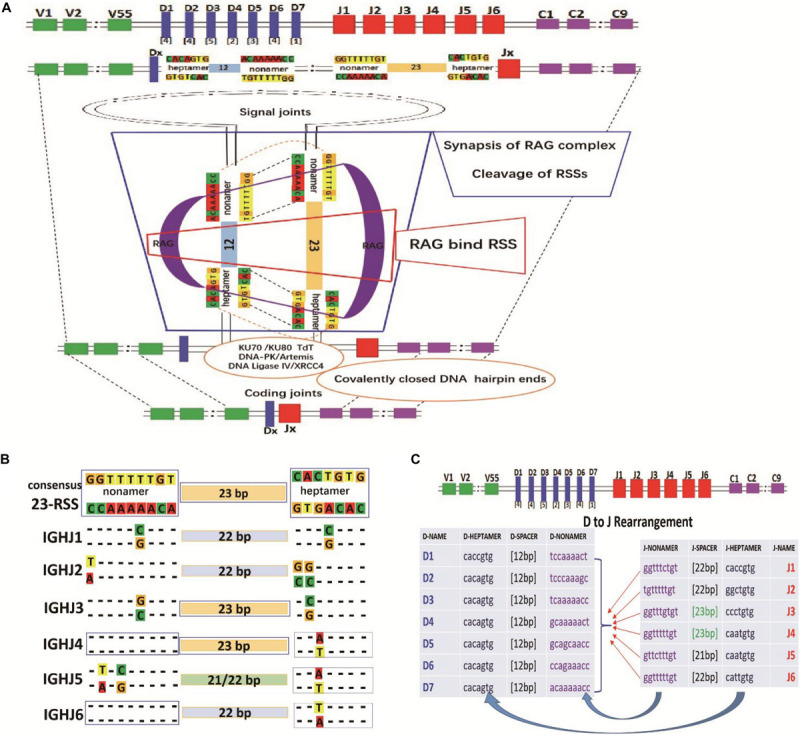
RSS composition characteristics during the IGHD-IGHJ recombination. **(A)** The schematic diagram of IGHJ and IGHD recombination. **(B)** The composition characteristics of human 9-23-7 RSSs (IGHJ-non-amer–IGHJ- spacer–IGHJ-heptamer). **(C)** The pairing of IGHJ (7-12-9) RSSs and IGHD (9-23-7) RSSs during the IGHD-IGHJ recombination.

Recombination of IGHJ-IGHD can be divided into two phases. The first phase involves recognition and cleavage of the DNA, and the second phase involves resolution and joining (Lewis, 1994; Bogue and Roth, 1996). In the evolutionary process, the human IGHJ non-amer sequence is 5′-GGTTTTTTT-3′ (the complementary sequences, CCAAAAAAA), and the IGHD non-amer sequence is 5′-ACAAACC-3′ (the complementary sequences, TGTTTTTGG). This evolutionary IGHD-IGHJ “double-stranded complementary pairing” relationship may play a role in the efficiency of D-J recombination. The IGHJ-IGHD recombination schematic diagram is shown in Figure 5A.

To investigate whether RSSs affect IGHJ usage, we obtained human IGHJ gene sequences (X97051, X86356, M25625, J00256, AJ879487 from the IMGT, and GenBank) for RSS composition analysis. The composition and characteristics of the human IGHJ RSSs [non-amer—spacer—heptamer (9-23-7)], J region sequence and AA are shown in Supplementary Tables 9–11. IGHJ4 and IGHJ6 have the consensus non-amer sequences “5′-GGTTTTTGT-3′” (the complementary sequence is “CCAAAAACA”). However, the non-amer had one or two base mutations in other IGHJ families. Position 4 of IGHJ1 mutated from A to G, position 9 of IGHJ2 mutated from C to A, position 4 of IGHJ3 mutated from A to C, position 6 of IGHJ5 mutated from A to G, and position 8 of IGHJ5 mutated from C to A (Supplementary Table 9 and Figure 5B). The consensus heptamer is CACAGTG/GTGTCAC. Position 5 of IGHJ4 and IGHJ5 mutated from G to T (IGHJ6 mutated to A), position 4 of IGHJ1 mutated from A to G, position 6 of IGHJ2 mutated from T&G to C, and position 6 of IGHJ3 mutated from T to G. In addition, IGHJ4 and IGHJ3 have a consensus spacer length (23 bp), while the spacer length is reduced by 1 or 2 bases in other IGHJ gene families (IGHJ1-22 bp, IGHJ2-22 bp, IGHJ5-21 or 22 bp, and IGHJ6-22 bp; Figure 5C).

Overall, compared to the conserved RSS, the IGHJ4 gene subfamily is roughly consistent, the spacer lengths are changed in IGHJ6, the non-amer and heptamer are altered in IGHJ3, the spacer lengths and the non-amer are changed in IGHJ5, and the non-amer, heptamer, and spacer lengths are changed simultaneously in IGHJ1 and IGHJ2. There were different code end sequences (AA) in the IGHJ genes IGHJ4 (15AA), IGHJ1 and IGHJ2 (17AA), IGHJ3 and IGHJ5 (16AA), and IGHJ6 (20AA).

## Discussion

The V(D)J gene family of the human BCR heavy chain variable region contains 56 functional V genes with 3′ ends of 7-23-9 RSS, 27 functional D genes with 3′ ends of 9-12-7 RSS and 5′ ends of 7-12-9 RSS, and 6 functional J genes with 5′ ends of 9-23-7 RSS. The recombination starts with recombination of the 3′ end of the D gene and the 5′ end of the J gene, and then the 3′ end of the V gene is recombined with the 5′ end of the D gene (D-J recombination). In the peripheral BCR-H repertoires, the usage frequency of each V(D)J gene is related to the preferred usage in the initial rearrangement, the selection of self-tolerance and the response of peripheral clonal proliferation. However, the mechanism and significance of differential selection among V(D)J gene subfamilies have not been fully elucidated (Lewis, 1994; Bogue and Roth, 1996; Murphy et al., 2014).

We investigated the usage frequency of the 6 IGHJ genes in unique BCR-H repertoires (in-frame and out-of-frame) by HTS under normal physiological and various pathological conditions. In addition, we analyzed non-HTS-derived BCR-H sequences from the IMGT database, the HTS-derived BCR-H sequences from the public database (other laboratory), and the usage frequency data of 6 IGHJ genes from 19 published articles. The results indicate that IGHJ4 has a significantly high usage frequency in all subjects, various tissues, and different B cell subset samples. IGHJ6, IGHJ3, and IGHJ5 have medium usage frequencies, and IGHJ1 and IGHJ2 have significantly low usage frequencies. Taken together, these results suggest that the recombination selection of each human IGHJ gene is non-random and rarely influenced by antigen selection, which is quite different from statistically expected recombinations.

### The IGHJ Non-amer and Recombination Frequency

Early studies suggested that the composition characteristics of human IGHJ RSSs may affect the usage frequency of IGHJ in the initial rearrangement. In 1987, Akira S et al. found that two sets of heptamer (CACTGTG) and non-amer (GGTTTTTGT) sequences were enough to initiate the V(D)J joining if the 12-bp and 23-bp spacer rule is satisfied in the recombination-competent pre-B cell line (Akira et al., 1987). A point mutation in the heptamer sequence or a change in the combination of the two spacer lengths (21 bp 22 bp 24 bp/11 bp 13 bp) would drastically reduce the recombination frequency.

Variation from the conserved sequences in the heptamer and non-amer of the RSSs is considered a major factor affecting the relative representation of gene segments in the primary repertoire. The mechanism of RSSs on gene recombination is mainly related to the interaction efficiency of RAG protein (recombinase; Difilippantonio et al., 1996; Ramsden et al., 1996; Spanopoulou et al., 1996; Akamatsu and Oettinger, 1998; Swanson and Desiderio, 1998). Based on the composition of human IGHJ gene families, we found differences in RSSs among 6 IGHJ gene families (Supplementary Table 9 and Figure 5), which suggests that these differences may affect the usage frequency (non-random) of IGHJ gene families.

The non-amer of human IGHJ4 and IGHJ6 is the conserved sequence 5′-GGTTTTTGT-3′ or 5′-CCAAAACA-3′, while the other IGHJ non-amers have one or two base mutations. Experiments *in vitro* based on B cell lines showed that the mutation of non-amer had a significant effect on the corresponding gene recombination. Ramsden DA et al. found that the non-amers were probably the most important element in initial RAG protein binding (Ramsden et al., 1996). A single base mutation of the non-amer resulted in a reduction in overall cleavage levels when the heptamer was retained, but the entire non-amer was substituted with random sequence. Both nicks and hairpins were still found, but overall cleavage was reduced fold. Kowalski D et al. found (Kowalski et al., 1988; Kowalski and Eddy, 1989) that A-rich core sequences of the non-amer may be important to facilitate strand dissociation during the process of recombination.

The presence of three consecutive A residues was necessary for efficient recombination in the non-amer; furthermore, the nucleotides flanking the A-rich core needed to be other than one residue. The mechanism may be that the recombinase must measure the distance between the heptamer and the non-amer to satisfy the 12/23-bp spacer rule (Hesse et al., 1987; Akamatsu et al., 1994; Ramsden et al., 1996; Steen et al., 1997; Larijani et al., 1999). Akamatsu et al. (1994) found that the A residue at position 5 (non-amer A-rich core) was most crucial in their recombination assay. However, Hesse et al. (1987) considered that the “A residue” at position 6 (non-amer) was most crucial in their recombination assay. Regarding the effect of non-amer A-rich core mutation and corresponding gene usage, Akamatsu et al. (1994) found that recombination frequency decreased to 27.3% of the control with the mutant 9-4G [position 4 of non-amer (number 9 represent non-amer) was changed to G, defined by IMGT]. A mutant at position 9-5C gave the lowest recombination frequency (10.4%). With the double mutant at positions 9-3G and 9-4G, the joining rate dropped only to 19.3% (9-6G and 9-7G was 26.0%). According to the results from cell line experiments, human IGH4 and IGHJ6 gene subfamilies appear to have a “complete A-rich core” in the non-amer (conserved), which may play an important role in their high usage selection. However, 9-4A of human IGHJ1 is mutated to 9-4G, 9-4A of IGHJ3 is mutated to 9-4C, 9-6A of IGHJ5 is mutated to 9-6G, and 9-8C is mutated to 9-8A, which is a possible cause of their disfavored usages.

In addition, Akamatsu et al. (1994) found that the non-amer 9-2C was changed to 9-2A, and the recombination frequency was reduced to 2.7% of the control level; 9-2C was changed to 9-2T, and the frequency was reduced to 12.9%; and 9-2C was changed to 9-2G, and the frequency remained at 61.3%. When 9-8C/9-9C were changed to 9-8N/9-9A, the recombination frequency dramatically dropped to less than 0.1%, which suggested that the C residue plays an important role when the recombinase measures the distance between the heptamer and the non-amer sequences. In this study, one factor for the low usage frequency of the human IGHJ2 gene may be its 9-9C mutation to 9-9A.

### The IGHJ Heptamer and Recombination Frequency

Human IGHJ4 and IGHJ5 genes have the same heptamer sequence (CAATGTG/GTTACAC). Position 7-3C is mutated to 7-3A compared to the conserved heptamer, and 7-3C is mutated to 7-3T in IGHJ6, while the heptamer sequences of the IGHJ4/IGHJ5/IGHJ6 gene subfamilies are uniform on the double strand. Position 7-4A is mutated to 7-4G in the IGHJ1 gene, position 7-6T/7-7G is mutated to 7-6C/7-7C in the IGHJ2 gene, and position 7-6T is mutated to 7-6G in the IGHJ3 gene.

The relationship between the heptamer and the recombination frequency of the corresponding gene family has been confirmed by several laboratories. Previous studies found that the mutation of the entire heptamer resulted in low levels of nicking distributed across several sites, the mechanism of heptamer affecting recombination was related to the formation of hairpins, and the nicks and hairpins were reduced 2-fold when the sequence of the last four positions of the heptamer was changed (Hesse et al., 1989; Ramsden et al., 1996). In addition, nicking formation depended on the heptamer for the generation of double strand breaks (DSBs) by RAG1 and RAG2, and the non-amer at the correct distance would improve heptamer efficiency in the natural RSSs. The first three nucleotide positions were nearly 100% conserved (CAC/GTG) in the BCR gene. The mutations were in the first three positions, and cleavage was impaired either at the nicking step or the hairpin formation site. No rearrangement was detected with the mutant at position l (7-1G). Mutations at position 2 (7-2T) and position 3 (7-3G) produced detectable levels of recombination, 0.5% and 0.6%, respectively. The G residue at position 5 was changed to C (7-5C), and the recombination frequency dropped to 5.9% of the control level. For the rest of the residues in the heptamer, mutation effects were moderate, ranging from 28.5 to 52.0%. Akamatsu Y et al. (Ramsden et al., 1996) found that no rearrangement was detected with the mutant at position l (7-1G), and mutations at position 2 (7-2T) and position 3 (7-3G) produced detectable levels of recombination, 0.5% and 0.6%, respectively. The recombination frequency dropped to 5.9% of the control level when the G residue at position 5 was changed to C (7-5C); for the rest of the residues in the heptamer, mutation effects were moderate, ranging from 28.5 to 52.0% (Akamatsu et al., 1994).

The first three positions of the 6 human IGHJ gene subfamily heptamers are a conserved CAC/GTG sequence. Based on the results of Akamatsu et al. (1994), position 7-4A of human IGHJ1 mutated to 7-4G, and 7-6T/7-7G of IGHJ2 mutated to 7-6C/7-7C, which may be one important factor causing their low usage frequency. In addition, the 7-5G mutation (IGHJ3, IGHJ4, IGHJ5, and IGHJ6) may have a moderate effect on their usage frequency. The effect of mutations in the human IGHJ heptamer on usage frequency needs to be further explored.

### The RSS Spacer and Recombination Frequency

The length of the spacer is also a determining factor contributing to the usage frequency of V(D)J rearrangement. Human IGHJ4 and IGHJ3 gene subfamilies have a conserved 23 bp length; however, the IGHJ1, IGHJ2, IGHJ5, and IGH6 gene subfamilies have 21 bp or 22 bp spacer lengths.

Akamatsu et al. (1994) found that the recombination frequency dropped to 7.7% with the 11-bp RSSs when one C residue was added to the 12 bp RSSs (13 bp spacer; 11.0% joining rate); when two C residues were added (14 bp spacer), recombination dropped below the detection level, indicating that RSS spacer length was critical for combination frequency. Nadel et al. (1998) found that the effect of the spacer on the recombination rate of various human Vk gene segments in the peripheral repertoire correlated with their frequency in pre-B cells (*in vivo*). Steen SB et al. found that changing the spacer length by one nucleotide (23 bp1 bp only moderately reduced DSB formation, altering the spacer length by greater than one nucleotide (23 bp-2 bp and 23 bp-3 bp), severely reduced cleavage to a lesser degree (Steen et al., 1997). If each RSS contains a severe mutation (12 bp-3 bp/23 bp-3 bp), no DSBs were observed. According to the above research, the length of the 23 bp spacer of the human IGHJ4 and IGHJ3 gene subfamilies is an important factor in the higher usage frequency, and the length reduction of the 23 bp spacer in the IGHJ1, IGHJ2, IGHJ5, and IGHJ6 genes reduces their recombination usage.

The sequences of RSSs may affect the usage frequency of V(D)J gene recombination. Fanning L et al. found that when the Igk 12 bp spacer of the natural sequence CTAC “A” GACTGGA was changed to CTAC “C” GACTGGA but the corresponding 23RSSs-GTAGTACTCCACTG TCTGGCTGT were not changed, the mutant proximal RSSs were consistently used less frequently (Fanning et al., 1996). In addition, the recombination efficiency was 63.0% of the control level when the 12 bp spacer was changed to an artificial sequence GATCGATCGATC (Akira et al., 1987; Hesse et al., 1989; Akamatsu et al., 1994). Larijani et al. (1999) found that the frequency of recombination decreased by approximately 5-fold when the V81x spacer (AGCAAAAGTTACTGTGAGCTCAA) was replaced by that of VA1 (TTGTAA CCACATCCTGAGTGTGT). Montalbano et al. (2003) found that single base pair changes in the spacer sequence can significantly affect recombination efficiency. Nadel et al. (1998) confirmed that natural variation in spacer sequences could contribute to the non-random use of human V genes observed *in vivo* and that a randomly generated variant of a human V spacer was significantly worse in recombination efficiency. These results suggest that the spacer sequence plays an important role in recombination efficiency. Our results show that the ratio of AT and CG in 23 bp spacer sequences of 6 human IGHJ gene families is inconsistent (Supplementary Table 9). Base C has the highest ratio in IGHJ4. Is this the reason for the higher usage frequency in the recombination of the IGHJ4 gene subfamily? Whether the base composition of spacer sequences such as the non-amer has the key “A-rich core” structure need to be further explored.

### Distance and Recombination Frequency

It has been confirmed that the proximal gene has preferred usage in the initial rearrangement (Yancopoulos et al., 1984; Perlmutter et al., 1985; Reth et al., 1986). Malynn et al. (1990) believe that the difference in IGHV gene usage in adult spleen B cells is mainly due to the selection of the initial rearrangement rather than the changes in expression frequency after rearrangement. The “proximal and distal” studies of BCR recombinant genes are mainly focused on the V gene. “Proximal and distal” differences in the J gene have not been reported. In our results, we did not find the “proximal” phenomenon in the 6 IGHJ gene families with high usage frequencies.

### Other Factors and Recombination Frequency

Ramsden et al. (1996) found that the sequence of the coding end may be related to the usage frequency of gene combination. We found that there are differences in the amino acid length and the coding flank sequences of the human 6 IGHJ families (Supplementary Table 9). The IGHJ4 gene has the shortest 16 amino acid components. The sequence of the coding end and AA length may affect the usage frequency of IGHJ. We analyzed the deletions of the 3′D end, the 5′J end and the insertion between the D-J end and found that there was a difference between the 5′J end of IGHJ4 and other IGHJ genes (Figure 3 and Supplementary Table 11). Whether it was a factor for high usage of IGHJ4 needs to be further studied. In addition, IGHD gene families may also affect the non-randomness of IGHJ genes. VanDyk LF et al. suggested that V(D)J recombination was targeted by RSSs, while the RSSs flanking D segments appeared to be equivalent. They were not randomly utilized, suggesting that the D-3′ RSSs were not simply superior targets for the D-J recombinase but instead that targeting certain 12/23-bp spacer RSS combinations is more effective (VanDyk et al., 1996).

We found that the conserved non-amer of IGHJ4 and IGHJ6 had a higher “double-stranded complementary paired” rate than the 27 IGHD non-amer sequences (Supplementary Table 10 and Supplementary Figure 1), although it did not show obvious differences. At present, no evidence to support that RAG has an effect on the “double-stranded complementary paired” of the J-heptamer to D-heptamer and J-non-amer to D-non-amer exists; the mechanism is still unknown. We hypothesize that two genes with high complementarity (7-7/9-9) may be more favorable for binding, cleaving, hairpin formation, and DSB in the recombination process (Figure 5), which is a very interesting entry point for further research in BCR gene recombination.

In summary, for the possible impacts of RSSs on different IGHJ usage frequencies, we analyzed the mutation data of RSSs from the literature. We found the changes associated with lengths of the IGHJ6 spacer (23 bp), the non-amer and heptamer of IGHJ3 and the non-amer of IGHJ5. However, the changes in the non-amer, heptamer and spacer of IGHJ1 and IGHJ2 were more significant. These may be factors that resulted in non-random usage of the human IGHJ gene (generally, IGHJ4 > IGHJ6 > IGHJ3> or ≈IGHJ5 > IGHJ2≈IGHJ1) in the initial rearrangement. Thus, RSSs may influence the initial human BCR-H repertoires (before antigen selection), and that the “background” repertoire of IGHJ genes (the initial usage frequency) is highly skewed. These results suggest that the information of background repertoire should be considered when analyzing the significance and mechanism of each IGHJ gene usage in self-tolerance selection and the clonal proliferative response.

## Materials and Methods

### Subjects

The subjects included six healthy volunteers (6 samples: H-1, H-2, H-3, H-4, H-5, and H-6; unpublished), two volunteers with systemic lupus erythematosus (SLE; including 6 total samples pretreatment, during treatment and after treatment, namely, S1-1, S1-2, S1-3, S2-1, S2-2, and S2-3; our published data, Shi et al., 2016), three volunteers with breast cancer (including 9 total samples pretreatment, during treatment and after treatment, namely, B3-1, B2-1, B1-1, B3-2, B2-2, B1-2, B3-3, B2-3, and B1-3), two volunteers with a high titer of HBsAb (2 samples: HBsAb-1, HBsAb-2; our published data, Pan et al., 2015) and three volunteers with samples before and after immunization with the HBV vaccine (6 IgM samples (V1-BM, V1-AM, V2-BM, V2-AM, V3-BM, and V3-AM) and 6 IgG samples (V1-BG, V1-AG, V2-BG, V2-AG, V3-BG, and V3-AG; our published data, Ma et al., 2017). The peripheral blood samples were obtained from the Affiliated Hospital of Zunyi Medical University. All the volunteers were informed of the purpose of peripheral blood collection and were under a protocol approved by The Committee on the Ethics of Human Experiments of Zunyi Medical University, and all the experiments were performed in accordance with the guidelines of the committee. Peripheral blood mononuclear cells (PBMCs) were obtained using Ficoll 1640 (Biochrom AG, Berlin, Germany) density centrifugation.

### Total RNA/DNA Extraction and cDNA Synthesis

Total RNA was extracted from the PBMCs in three volunteers with immunization with HBV vaccine according to the manufacturer’s protocol for the total RNA extraction kit (OmegaBio-Tek). The total RNA was then reverse transcribed into cDNA using Oligo dT18 according to the manufacturer’s protocol for the reverse transcription kit (MBI, Fermentas). The genomic DNA from PBMCs in other samples was obtained using the QIAamp DNA Mini Kit (QIAGEN, CA) and was stored in a QIAsafe DNA tube (QIAGEN).

### High-Throughput Sequencing

Before HTS, the concentration and purity of DNA or cDNA of samples need to reach the requirement of BCR CDR3 sequencing and the volume of every sample was the same (the whole total DNA or cDNA needs to reach 2 μg). Multiplexed PCR amplification is performed to amplify rearranged CDR3 sequences, designing an upstream primer and downstream primer in the VH functional gene region and JH functional gene region, respectively. Every primer was set in the specific site of BCR H chain. All the DNA samples were sent to Adaptive Biotechnologies Corp^1^ for multiplex PCR amplification of human BCR-HCDR3 regions. Error from bias in this multiplex PCR assay was controlled using synthetic templates (Carlson et al., 2013), and the HCDR3 sequences were acquired by HTS on the ImmunoSEQ platform (see text footnote 1, respectively; Pan et al., 2015). All the PCR products of cDNA samples after PCR amplification were sent to Tongji-SCBIT Biotechnology Corporation for HTS, and detailed experimental procedures have been described in our previous article (Ma et al., 2017). The HCDR3 regions were identified within the sequencing reads according to the definition established by the International ImMunoGeneTics Information System (IMGT)^2^. IMGT numbering (Lefranc et al., 2003, 2005; Lefranc, 2011) was used to identify which V(D)J segments contributed to each HCDR3 sequence.

### Public Data

We used 9,340 unique in-frame BCR-H sequences (non-HTS data in different pathological states) derived from the IMGT/LIGM-DB^3^ to analyze the IGHJ gene frequency by IMGT/HighV-QUEST (Shi et al., 2014). These in-frame sequences have been deposited in the IMGT/LIGM-DB over the past few decades and come from different laboratories [IMGT generally does not store out-of-frame sequences, therefore, analysis involving IMGT data only includes in-frame data (Figure 1C and Supplementary Table 3)]. To some extent, these sequences represent a data set under multiple conditions. To show the results at different depths, high depth HCDR3 sequences of naive B cells (PLOS-1: *n* = 48,167) and memory B cells (PLOS-2: *n* = 50,290) from a public database (single well sequencing data from 1 healthy volunteer) were used for this study (DeWitt et al., 2016). The unique in-frame BCR-H sequences (*n* = 84,804) and out-of-frame sequences (*n* = 13,653) were compared and analyzed by IMGT/HighV-QUEST software in this study.

### Sequence Analysis

The raw sequences in FASTA format were analyzed with IMGT/HighV-QUEST online software (version 1.3.1, http://www.imgt.org). Using the IMGT summary document, the sequences not meeting the following criteria were filtered out: (1) no results (sequences for which IMGT/HighV-QUEST did not return any result) and (2) unknown [sequences for which no functionality was detected. This category corresponds to the sequences for which the junction could not be identified (no evidence of rearrangement, no evidence of junction anchors)]. In-frame and out-of-frame unique sequences remaining after filtering were used for IGHJ gene frequency, D-J pairing, and nucleotide insertion and deletion analyses. All samples data of each group were used for analysis, and we did not separately analyze the data before and after different interventions.

### RSS Composition Analysis

According to the accession numbers of these human IGHJ and IGHD genes in IMGT/LIGM-DB and GenBank, we obtained detailed annotations of complete human genome sequences for RSS composition analysis, including sequence characteristics of non-amers and heptamers, length characteristics of 12 bp and 23 bp spacers, and the IGHJ gene segment (amino acid) composition of code end.

### Software and Statistics

IMGT/HighV-QUEST (version 1.3.1) was used for identification of sequences (IGHJ and IGHD genes), evaluation of functionality and statistical analysis of the sequence data; IMGT/V-QUEST (version 3.3.1) was used for identification of non-amers, heptamers, 12 bp and 23 bp spacers, and IGHJ gene segments of the coding end; Microsoft Office Excel (version 365) was used for storage, filtering and statistical analysis of the sequences. The resulting sequences were graphed using Prism 8 software (GraphPad). IGHJ gene usages and Insertions and deletions of the nucleotides were compared using one-way ANOVA with Bonferroni correction, respectively. All statistically significant differences are indicated as ^∗^*p* < 0.05; ^∗∗^*p* < 0.01, and ^∗∗∗^*p* < 0.001.

## Data Availability Statement

All datasets generated for this study are included in the article/Supplementary Material.

## Ethics Statement

The studies involving human participants were reviewed and approved by Zunyi Medical University. The patients/participants provided their written informed consent to participate in this study. The animal study was reviewed and approved by Zunyi Medical University.

## Author Contributions

XY designed the research and wrote the manuscript. BS, XD, and QM analyzed the data. JY and BS did the SLE volunteers experiment. LM did the healthy volunteers experiment and analyzed parts of the data. SS did the breast cancer volunteers experiment. XW did the HBV vaccine volunteers experiment. JP did the high HbsAg volunteers experiment. XH and DS analyzed the part of data. All authors read and approved the final manuscript.

## Conflict of Interest

The authors declare that the research was conducted in the absence of any commercial or financial relationships that could be construed as a potential conflict of interest.

## Footnotes

1 http://www.adaptivebiotech.com

2 http://www.imgt.org

3 http://www.imgt.org/ligmdb
